# Pilomatrixoma of the medial arm presenting as an indeterminate soft tissue lesion on MRI: A case report

**DOI:** 10.1016/j.jpra.2026.10.001

**Published:** 2026-10-14

**Authors:** Edward T.C. Dong, Aksel N. Olsen, Benjamin Perone, Matteo Scampa, Daniel F. Kalbermatten, Jérôme Martineau

**Affiliations:** Department of Plastic, Reconstructive, and Aesthetic surgery, Geneva University Hospitals, Geneva University, Geneva, Switzerland

**Keywords:** Pilomatrixoma, Soft tissue lesion, Cutaneous tumor, Imaging

## Abstract

Pilomatrixoma is a benign hair follicle matrix cell tumor and one of the most common cutaneous tumors in children and young adults. We present the case of a 33-year-old male evaluated for a progressively enlarging, pruritic subcutaneous nodule on the medial right arm. Multimodal imaging, including ultrasonography and magnetic resonance imaging (MRI), revealed an indeterminate, hypervascular dermal mass measuring 8 × 17 × 11 mm with hypodermal extension. Given the ambiguous radiological features and increasing functional discomfort, the patient underwent an elective excisional biopsy under local anesthesia approximately five months after the initial ultrasound. Histopathological analysis established the definitive diagnosis of a 1.7 cm pilomatrixoma characterized by focal induration and microcalcifications. The postoperative course was uneventful. This case highlights the diagnostic limitations of cross-sectional imaging for atypical superficial soft-tissue masses and reinforces the necessity of surgical excision for definitive histopathological characterization.

## 1. Introduction

Pilomatrixoma is a benign skin appendage tumor arising from the matrix cells of the hair follicles. Although it is considered uncommon, it is one of the most frequently encountered cutaneous tumor in children and young adults with a marked female predominance.^1^

Pilomatrixoma typically presents as a solitary, firm, mobile, subcutaneous nodule, usually covered by normal or occasionally discolored overlying skin. Intertumoral calcifications are frequently observed. These lesions most commonly arise in the head and neck region and the upper extremities, although they may occur at any anatomical site containing hair follicles.^2^ Despite its benign nature, pilomatrixoma is frequently misdiagnosed preoperatively, most commonly as an epidermoid or dermoid cyst, owing to its heterogeneous clinical presentation and the absence of pathognomonic clinical features.

## 2. Case presentation

A 33-year-old male patient with no significant medical history was referred by his general practitioner to our clinic for evaluation of a progressively enlarging pruritic subcutaneous nodule on the medial aspect of the right arm, causing increasing functional discomfort. On clinical examination, a mobile subcutaneous mass of approximately 2 cm was palpable on the medial aspect of the right arm, with overlying erythema and skin thinning. Because of the raised appearance and its nipple-like appearance and the location near the axillary extent of the mammary ridge, one of the initial hypotheses was a potential accessory nipple, although the medial arm lies outside the usual course of the mammary ridge and the nodule was subcutaneous and mobile (Fig. 1).

**Fig. 1:**
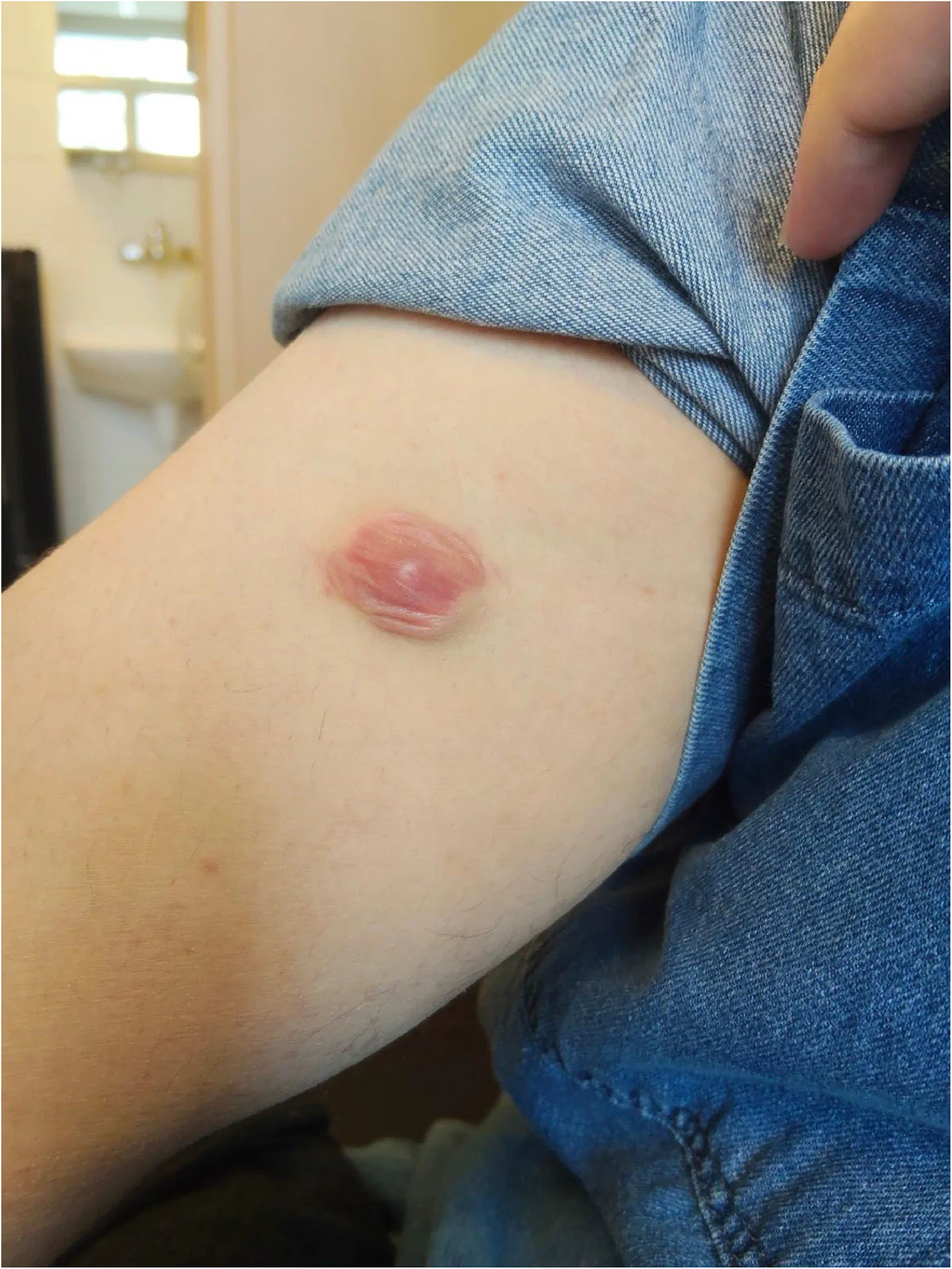
Preoperative clinical photograph of the lesion on the medial aspect of the right arm.

Pre-referral workup included soft tissue ultrasound, performed approximately five months prior to our initial consultation, which showed a well-demarcated subcutaneous lesion with heterogeneous content, no liquid component, and Doppler hypervascularization, without a definitive diagnosis (Fig. 2). Following our consultation and given the indeterminate nature of the lesion, with a mass of increasing size, complementary magnetic resonance imaging (MRI) of the skin and soft tissues was requested.

**Fig. 2:**
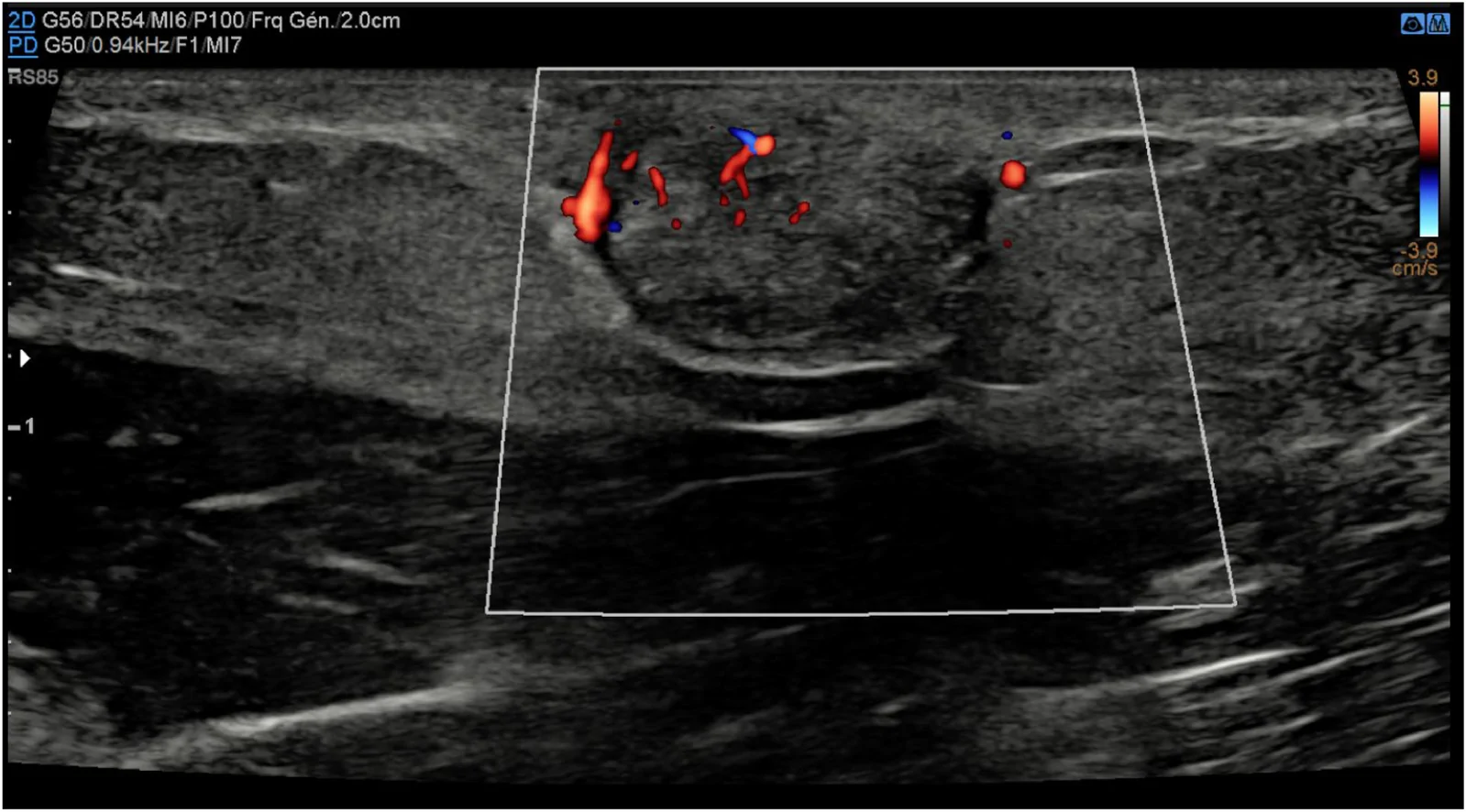
Ultrasound showing a well-demarcated subcutaneous lesion with heterogeneous echogenicity, no fluid component, and Doppler hypervascularization.

MRI identified a dermal nodule on the medial aspect of the right arm measuring 8 × 17 × 11 mm, heterogeneous and hypointense on T1-weighted and fat-saturated proton-density sequences, with heterogeneous enhancement and a small cystic component (Fig. 3). The MRI showed the lesion extended to the hypodermis, with a preserved fat plane of 1 mm from the superficial fascia of the triceps muscle, and no evidence of deep muscular invasion. The MRI concluded a dermal lesion with hypodermic extension of indeterminate etiology, recommending correlation with excisional biopsy.

**Fig. 3:**
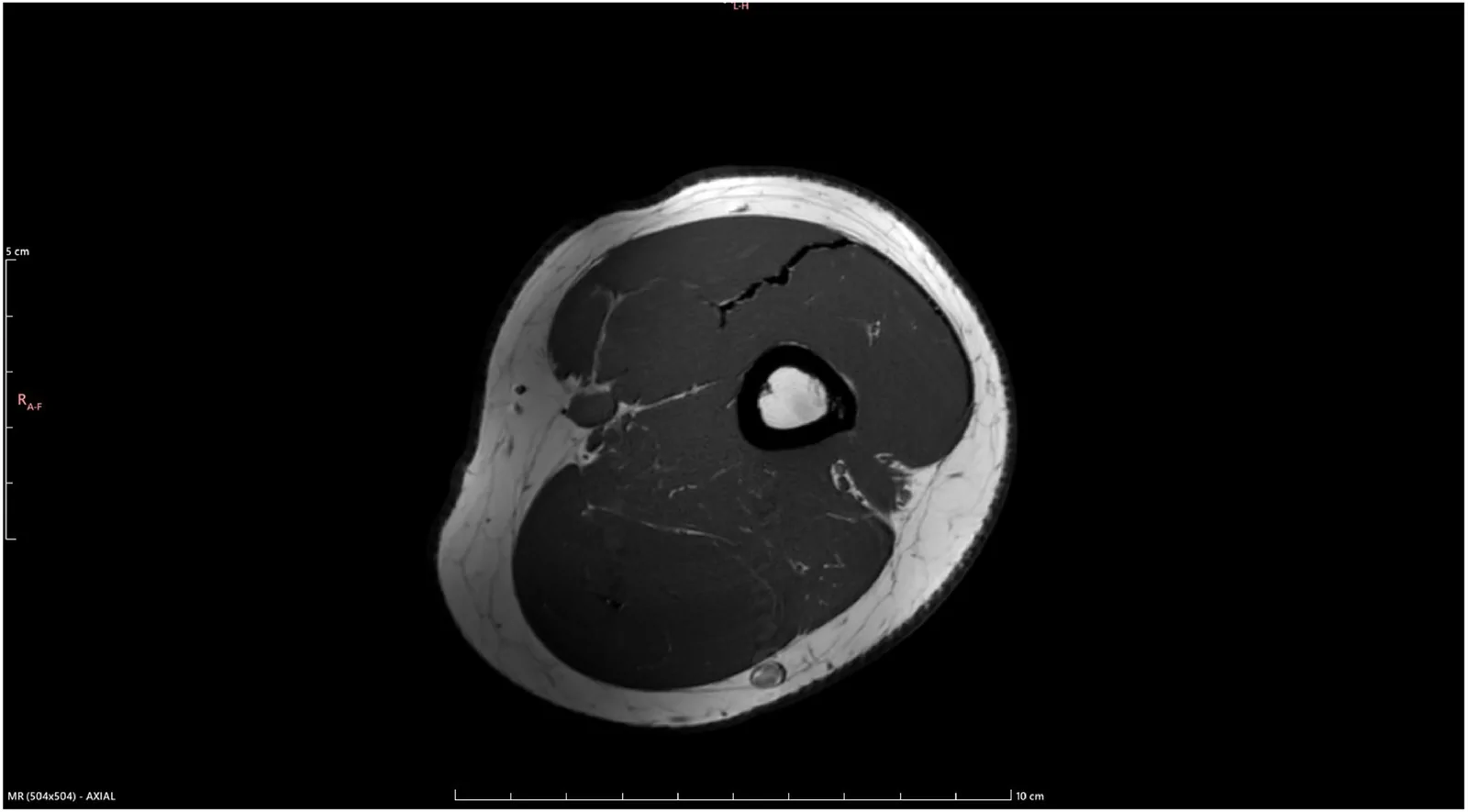
MRI demonstrating a dermal nodule on the medial aspect of the right arm measuring 8 × 17 × 11 mm, heterogeneous and hypointense on T1-weighted and fat-saturated proton-density sequences, with heterogeneous enhancement and a small cystic component. The nodule extends into the subcutaneous tissue while maintaining a 1 mm fat plane from the superficial fascia of the triceps muscle, with no evidence of deep muscular invasion.

Excisional biopsy was performed as an elective ambulatory procedure under local anesthesia. An elliptical incision was made according to preoperative markings overlying the nodule. Dissection was carried out using Metzenbaum scissors. The nodule, which demonstrated a calcified appearance during intraoperative inspection (Fig. 4). Both the skin ellipse and the nodule were sent for histopathological analysis. Meticulous haemostasis was achieved and the wound irrigated with normal saline. Wound closure was performed in two layers using interrupted inverted absorbable sutures (Monocryl 3-0; Ethicon Inc., USA) followed by a continuous intradermal running suture (Monocryl 4-0; Ethicon Inc., USA).

**Fig. 4:**
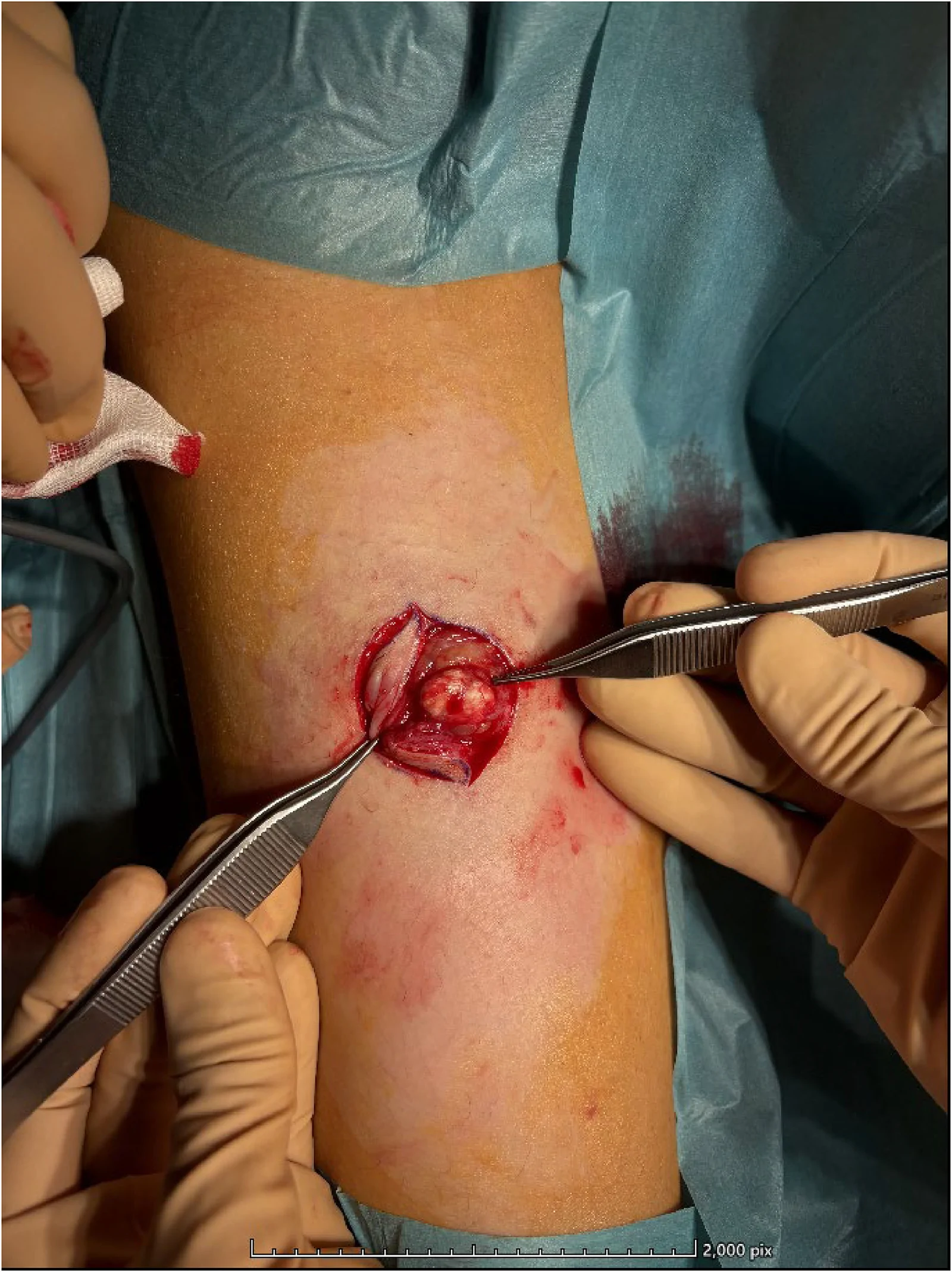
Intraoperative photograph showing the calcified aspect of the lesion.

Histopathological analysis confirmed a pilomatrixoma measuring 1.7 cm in greatest dimension, with a smooth beige-whitish surface, focal induration, and microcalcification. The overlying skin ellipse (3.6 × 1.2 cm) was free of lesion. The local induration within the pilomatrixoma may have explained the redness of the skin as it may have caused irritation. The postoperative course was uneventful, and the wound was healed at the two-week postoperative follow-up.

## 3. Discussion

Pilomatrixoma was first described in 1880 by Malherbe and Chenantais.^3^ In a review including 2031 patients, there was a predominance of female patients, and most lesions occurred during the first and second decades of life, with a mean age of 16 years.^4^ It should be noted that pilomatrixoma can present at any age and that a second peak is usually observed in the sixth decade.^5^^,^^6^ Adult male presentation, as in the present case of a 33-year-old male patient, represents a minority of reported cases, which may contribute to diagnostic uncertainty.

The variable imaging appearance of pilomatrixoma represents a significant diagnostic challenge, as it can mimic a wide range of benign and malignant soft tissue tumors.^4^ In the present case, neither ultrasound nor MRI allowed definitive characterization of the lesion and neither modality ultimately changed management. The MRI findings are non-specific and have been reported in association with epidermal inclusion cysts, dermatofibrosarcoma protuberans, and other soft tissue neoplasms. The presence of calcifications, while characteristic of pilomatrixoma, are not always appreciable on MRI and may contribute to an indeterminate radiological appearance. Diffusion-weighted imaging (DWI) did not provide additional discriminatory value in this case, likely reflecting the heterogeneous, partly calcified/keratinous composition of pilomatrixoma rather than a technical limitation related to lesion depth, since DWI has been shown to reliably improve diagnostic accuracy for superficial soft-tissue masses in general.^7^

Clinical examination and biopsy remain the cornerstone of skin lesion management. Imaging is most useful when it can be expected to change the surgical plan, for instance in lesions that are large, deep, fixed to underlying structures, or clinically suspicious for malignancy, where it helps define the extent of the lesion, its relationship to the fascia and neurovascular structures, and the need for an extended excision. In the present case, the MRI mainly served to exclude deep extension, which clinical examination had already made unlikely. In retrospect, an excisional biopsy at the first consultation would have been a reasonable option and would have shortened the diagnostic pathway.

The present case deviates from the typical clinical presentation. The patient was a 33-year-old male, with the lesion showing cutaneous erythema and skin thinning, features more commonly associated with inflammatory or malignant processes than with a benign subcutaneous tumor.^2^^,^^5^ The associated pruritus and progressive enlargement also contributed to diagnostic uncertainty. All in all, these atypical features illustrate how pilomatrixoma can elude clinical recognition and emphasize the importance of a broad differential diagnosis when evaluating indeterminate subcutaneous nodules.

Surgical excision remains the gold standard treatment for pilomatrixoma, providing both definitive diagnosis and cure. Complete removal is essential, as incomplete one is associated with local recurrence, reported in approximately 2–3% of cases. The intraoperative finding of a calcified nodule was consistent with the final histopathological diagnosis and is a well-recognized macroscopic feature of pilomatrixoma. Malignant transformation to pilomatrix carcinoma, while exceedingly rare, has been reported and further supports the recommendation for complete surgical excision and histopathological confirmation in all cases.^4^

## 4. Conclusion

This case highlights that pilomatrixoma can present atypically, as an indeterminate soft tissue mass on ultrasound and MRI, mimicking more concerning neoplastic processes. Excisional biopsy was both diagnostic and curative, underscoring the importance of considering pilomatrixoma in the differential diagnosis of indeterminate subcutaneous nodules of the upper extremity in adults when imaging is non-specific.

## Patient written consent

The patient provided written consent for the use of anonymized clinical data.

## Declaration of competing interest

Daniel F. Kalbermatten is the Editor in Chief of JPRAS Open and Deputy Editor for the JPRAS, he was not involved in editorial review or the decision to publish this article. Other authors declare that they have no known competing financial interests or personal relationships that could have appeared to influence the work reported in this article.
